# Characteristics of participants in the first fully online National Diabetes Prevention Programme: A quantitative survey

**DOI:** 10.12688/hrbopenres.13807.3

**Published:** 2024-12-17

**Authors:** Clair Haseldine, Gráinne O'Donoghue, Patricia M Kearney, Fiona Riordan, Margaret Humphreys, Liz Kirby, Sheena M. McHugh

**Affiliations:** 1 Department of Public Health, University College Cork, Cork, County Cork, Ireland; 2 School of Public Health, Physiotherapy and Sports Science, University College Dublin, Dublin, Leinster, Ireland; 3 Health Service Executive, Cork, Ireland

**Keywords:** Diabetes prevention programme, Prediabetes, Psychosocial, Participation, Online, Quantitative survey, Participant characteristics

## Abstract

**Background:**

Diabetes prevention programmes (DPPs) are being implemented around the world to tackle the rise in type 2 diabetes. In 2021/22, the Health Service Executive(HSE) in Ireland piloted a fully online national diabetes prevention programme(NDPP). Characteristics and factors affecting participation may be different among people attending online DPPs compared to face-to-face programmes. The aim of this study was to describe the demographic, psychosocial and health characteristics of participants in the pilot of the online NDPP in Ireland.

**Methods:**

A survey from the evaluation of the English NDPP was adapted for the Irish context with Patient and Public Involvement (PPI) input. The survey was sent (between April and June 2022) to all individuals who attended the initial assessment of the pilot NDPP (n=73). It contained questions on health status, co-morbidities, motivation to improve health, quality of life, self-efficacy, beliefs about the risk of diabetes, participation(e.g. recollection and understanding of invite, number of sessions attended), as well as demographic information.

**Results:**

Response rate was 30.5% (n=22). Mean age of responders was 62 years (range 36–82 years) and over half were men (57.1%, n=12). The majority (81%, n=17) had attended 6 or more of the 14 sessions. Most (90.5% n=19) reported having family members or acquaintances with diabetes, had positive views of their current health status and high quality of life scores (71.4%, n= 15). Mental health scores were slightly higher than the national average. Over half (57.2%, n=12) were confident or very confident about participating in an online DPP. Almost all (95.2%, n=20) believed it was important to manage their risk of type 2 diabetes.

**Conclusions:**

Participants in the online pilot NDPP had positive views of their general health and positive psychosocial characteristics affecting their decision to participate. These beliefs may be modifiable intervention targets to encourage participation among non-attenders in future programmes.

## Introduction

Type 2 diabetes is a growing global health problem which can result in serious chronic health complications for individuals and places a financial burden on health systems ^1^.

Diabetes prevention programmes (DPPs) are being implemented worldwide to tackle the growing problem of type 2 diabetes ^2– 4^. High levels of participation in these programmes are essential to reduce the rates of people developing diabetes, however, a systematic review of DPPs in real world settings found low participation rates in 71% of programmes studied ^5^. A 2022 systematic review and meta synthesis of factors affecting lifestyle change in people with prediabetes found the individual’s evaluation of the importance of making lifestyle changes and the presence of supportive family and programmes facilitated change ^6^. Psychosocial factors (belief in the seriousness of type 2 diabetes, their elevated risk, and confidence the DPP could reduce their risk) were shown to be strongly associated with participation in a recent study examining uptake in the English NHS DPP ^7^. Practical barriers such as lack of time were also found to impact participation in a 2017 systematic review of diabetes prevention in primary care ^8^.

DPPs which are delivered synchronously online have been shown to address some of the barriers of face-to-face delivery while retaining effectiveness ^9^. Synchronous online programmes are delivered through videoconferencing with a group of participants and educators in real time. The NHS refer to this mode of delivery as *remote* ^10^ and in the US the Centre for Disease Control refers to it as *distance learning* ^11^. People attending such online DPPs may have different characteristics and different factors affecting their decision to participate compared to face-to-face programmes. For example, a greater proportion of men and more people of working age have been found to take part in synchronous online DPPs ^9^. However, very little is known about the psychosocial factors affecting participation. A recent paper exploring participation in the NHS digital DPP had 4 interviewees who declined the digital app in favour of the synchronous online programme as they preferred the peer support provided by the group format ^10^.

In Ireland, the Health Service Executive (HSE -national publicly funded healthcare system) piloted the National Diabetes Prevention Programme (NDPP) from June 2021 to September 2022. Prior to this there was very limited availability to short prediabetes courses that were not standardised. The Irish DPP was developed and delivered to a group synchronously online due to Covid 19 restrictions. This programme is novel and unique as the first NDPP to be developed specifically for the online format and offered exclusively online. No face-to-face version of the NDPP was available at the time. In other countries programmes were initially offered face-to-face and subsequently developed into programmes for digital or online delivery ^12, 13^. This study aims to describe the demographic, psychosocial and health characteristics of participants in the pilot of the online NDPP in Ireland. Understanding the reasons people participate in DPPs is important to help design strategies to improve the uptake, and therefore the effectiveness, of these programmes.

## Methods

This quantitative study used a postal survey for data collection. STROBE reporting guidelines were followed (Extended Data-Appendix 1). Ethical approval for the study was given by the Clinical Research Ethics Committee of the Cork Teaching Hospitals (ref: ECM 4 (n) 10/8/21).

## Materials

The survey was adapted from the survey used in the evaluation of the (NHS) DPP in England ^7^. The NHS survey was developed to identify factors associated with the uptake of the face-to-face DPP. The NHS survey was theory-based and included questions with established validity used in other studies where available. Adaptations to the survey for our study included using the Irish national census categories for ethnicity, using the Irish levels of education, using the full quality of life measure ^14^ and a full health confidence measure ^15^. As the Irish DPP is delivered entirely online, a question regarding confidence in participating in an online programme was added. Our survey was piloted prior to use with the research team, health professionals, a Patient and Public Involvement group (PPI) based at University College Cork and a university statistician was consulted. The PPI group consisted of 10 people with an interest in diabetes prevention. They advised on recruitment and the wording of questions. Based on their feedback on recruitment, the cover letters used the participant’s name rather than a generic address. Data collected included factors that could influence participation in the programme including health status, motivation to improve health, quality of life, self-efficacy, beliefs about the risk of diabetes, presence of co-morbidities as well as demographic and programme participation information (Extended Data-Appendix 2).

## Survey domains

### Participation

Recruitment for the NDPP pilot was from existing dietetic waiting lists and by referrals from GPs who had been informed of the pilot. Educators (dietitians in the HSE) invited people eligible to participate in the NDPP by phone. NDPP participants attended an initial assessment (in person, online or on the phone to have baseline measurements recorded, to receive a personal plan for lifestyle change and onboarding for the online session), and this was followed by 14 sessions of the NDPP (Extended Data-Appendix 3). Questions on participation in the NDPP included: recollection and understanding of invite, whether survey respondents were currently attending, and how many sessions they attended.

### Demographics

Questions on age, self-reported gender, and ethnicity as well as living status (living with how many others), employment status and highest level of education attained were included.

### Health factors

Health literacy was assessed using the Single Item Literacy Screener ^16^ and general health using the single general health item from the RAND SF-36 ^17^. Mental health was assessed using the Mental Health Inventory short 5 item scale (MHI 5) ^18^. This scale has 5 questions with 6 possible responses which are transformed into a score from 0 – 100 using a standard linear calculation where 100 represents optimal health. Quality of life was assessed using the EUROHIS-QOL 8-item index, which is a shortened version of the World Health Organisation Quality of Life Instrument- Abbreviated Version ^14^. It includes 2 questions each in psychological, physical, social and environmental domains with 5 possible responses and the overall score is calculated by summing up the responses. The maximum score is 40 with higher scores indicating higher quality of life. Other factors which could affect participation were investigated using questions on language and culture, disability, confidence in participating in an online programme and whether people had joined another lifestyle programme to improve their health in the past.

### Psychosocial factors

Health confidence was assessed using a 4 question Health Confidence Score ^15^. Responses were allocated a score from 0 = disagree to 3 = strongly agree. The summary score which ranges from 0 –12 was transformed to a linear scale from 0 – 100 with higher scores indicating higher health confidence. Self-efficacy was measured using a 4 item self-efficacy scale with 4 possible responses ^19^. The responses were summed up to give a score of 4 – 16, then converted to a 0 – 100 scale with higher scores indicating a higher self-efficacy. The remaining psychosocial questions examined the perceived need for the programme, the respondent’s vulnerability to developing diabetes, their ability to reduce the risk, how seriously they viewed the disease and how important it was to reduce the risk.

The 5 category Likert scale variables are combined to 3 and 4 categories in Table 1 and Table 2 for ease of presentation.

**Table 1.** Health characteristics of the survey sample.

	All N=21	Women N=9	Men N=12
	N (%)	N(%)	N(%)
**Health literacy** Help understanding written material			
Never	8 (38.1)	5 (55.6)	3 (25)
Rarely	8 (38.1)	2 (22.2)	6 (50)
Sometimes	4 (19)	1 (11.1)	3 (25)
Often	1 (4.8)	1 (11.1)	0 (0)
**General health**			
Excellent/ very good	2 (9.6)	0 (0)	2 (16.6)
Good	15 (71.4)	7 (77.8)	8 (66.7)
Fair	3 (14.3)	2 (22.2)	1 (8.3)
Poor	1 (4.8)	0 (0)	1 (8.3)
MHI 5 (0 – 100) *			
Mean (SD)	77.71 (12.1)	74.67 (10.2)	80 (13.32)
Range	52 – 96	60 – 80	52 – 96
**EUROHIS-QOL 8-item (8 – 40 scale) ^†^**			
Mean (SD)	31.38 (3.8)	30 (3.66)	32.33 (3.77)
Range	22 – 36	22 – 35	23 – 36
**Know someone with diabetes**			
Yes	19 (90.5)	9 (100)	10 (83.3)
No	1 (4.8)	0 (0)	1 (8.3)
Unsure	1 (4.8)	0 (0)	1 (8.3)
**Family member with diabetes**			
Yes	11 (52.4)	4 (4.44)	7 (53.8)
No	10 (47.6)	5 (55.6)	5 (41.7)
**Joined another group for improving health in the past**			
Yes	6 (28.6)	5 ( 55.6)	1 (8.3)
No	15 (71.4)	4 (44.4)	11 (91.7)
**Confidence joining online programme**			
Very unconfident/ unconfident	5 (23.9)	1 (11.1)	4 (33.4)
Neither confident nor unconfident	4 (19)	3 (33.3)	1 (8.3)
Confident / very confident	12 (57.2)	5 (55.6)	7 (58.3)
**Difficulties with health services due to language or** **culture**			
Agree/ agree strongly	0 (0)	0 (0)	0 (0)
Disagree/ disagree strongly	20 (95.2)	8 (88.9)	12 (100)
Missing	1 (4.8)	1 (11.1)	0 (0)
**Other health problems or disability more of a priority**			
Agree/ agree strongly	6 (28.6)	3 (33.3)	3 (25)
Disagree/ disagree strongly	13 (61.9)	4 (44.4)	9 (75)
Missing	2 (9.6)	2 (22.2)	0 (0)

Abbreviations: MHI-5 = Mental Health Inventory-5, QOL = Quality of Life *MHI-5 scored from 0 – 100 where 100 represents optimal health. ^†^EUROHIS-QOL 8-item index maximum score is 40 with higher scores indicating higher quality of life.

**Table 2.** Psychosocial characteristics of the survey sample.

	All N=21	Women N=9	Men N=12
	N (%)	N(%)	N(%)
**Health Confidence (0–100) ^‡^**			
Mean (SD)	66.6 (17.93)	62.96 (23.61)	68.89 (12.76)
Range	17 –100	17 – 100	50 – 100
**Self- efficacy (0–100) ^§^**			
Mean (SD)	67.08 (16.33)	61.08 (19.58)	71.5 (12.58)
Range	25 – 100	25 – 83	58 – 100
**Beliefs about the programme**			
The programme can help me reduce my risk of diabetes			
Agree/ agree strongly	21 (100)	9 (100)	12 (100)
Disagree/ disagree strongly	0 (0)	0 (0)	0 (0)
I can look after my risk without the programme			
Agree/ agree strongly	3 (14.3)	0 (0)	3 (25)
Disagree/ disagree strongly	18 (85.7)	9 (100)	9 (75)
The DPP couldn’t tell me anything new			
Agree/ agree strongly	0 (0)	0 (0)	0 (0)
Disagree/ disagree strongly	21 (100)	9 (100)	12 (100)
**Attitudes about the risk of diabetes**			
My risk of developing diabetes is too low to worry about			
Agree/ agree strongly	0 (0)	0 (0)	0 (0)
Disagree/ disagree strongly	21 (100)	9 (100)	12 (100)
If I carry on as normal, there is a good chance that I will develop diabetes			
Agree/ agree strongly	18 (85.7)	8 (88.9)	10 (83.3)
Disagree/ disagree strongly	2 (9.5)	0 (0)	2 (16.7)
Missing	1 (4.8)	1 (11.1)	0
Diabetes is not a very serious illness			
Agree/ agree strongly	0 (0)	0 (0)	0 (0)
Disagree/ disagree strongly	20 (95.2)	9 (100)	11 (91.7)
Missing	1 (4.8)	0 (0)	1 (8.3)
It is too difficult for me to change my lifestyle to reduce my diabetes risk			
Agree/ agree strongly	1 (4.8)	1 (11.1)	0 (0)
Disagree/ disagree strongly	20 (95.2)	8 (88.9)	12 (100)
Nothing I do can reduce my risk of getting diabetes			
Agree/agree strongly	0 (0)	0 (0)	0 (0)
Disagree/disagree strongly	21 (100)	9 (100)	12 (100)
I can do whatever is needed to reduce my risk of getting diabetes			
Agree/agree strongly	18 (85.7)	7 (77.8)	11 (91.7)
Disagree/disagree strongly	3 (14.3)	2 (22.2)	1 (8.3)
**Motivation to reduce risk**			
I am happy with my lifestyle as it is			
Agree / agree strongly	15 (70.4)	4 (44.4)	11 (91.7)
Disagree/ disagree strongly	5 (23.8)	4 (44.4)	1 (8.3)
Missing	1 (4.8)	1 (11.1)	0 (0)
It is important that I manage my risk of getting diabetes			
Agree/ agree strongly	20 (95.2)	9 (100)	11 (91.7)
Disagree/ disagree strongly	1 (4.8)	0 (0)	1 (8.3)
Going to this programme requires a lot of effort			
Agree/ agree strongly	1 (4.8)	0 (0)	1 (8.3)
Disagree/ disagree strongly	20 (95.2)	9 (100)	11 (91.7)

^‡^Health confidence scored from 0 – 100 with higher scores indicating higher health confidence. ^§^Self-efficacy scored from 0 – 100 with higher scores indicating higher self-efficacy.

## Participants and procedure

Of the 158 people invited to attend the NDPP, 73 attended the initial assessment and 67 went on to attend at least one further session. All those who attended the pilot of the NDPP for initial assessment (n=73) were sent an invitation to take part in the study, a survey, a participant information leaflet and a consent form by programme educators (Extended Data-Appendix 2, 4 – 6). For eligibility criteria for the NDPP see appendix 3. Invitations were sent by post between April and June 2022. Respondents had the opportunity to attend the programme for at least 7 months when they received the survey. A €10 voucher was offered to compensate people for the time spent completing the survey. Respondents returned the survey with the consent form directly to the researcher (CH) either via email or by post.

## Data analysis

To ensure data accuracy, 2 researchers (CH, ROM) entered the data from the surveys separately using SPSS software and then compared the 2 datasets to check for errors. Descriptive statistics were then used to summarise the data. Men's and women’s responses are also presented separately.

## Results

A total of 22 surveys were returned (30.5% response rate). One person did not sign the consent form and did not provide contact details, therefore, was excluded from the analysis. For a description of the NDPP and characteristics of those who attended the NDPP initial assessment see extended data Appendix 3 and 7.

### Participation in the NDPP pilot

All those that responded remembered being invited to the NDPP and almost all (90.5%, n=19) understood why they were invited. At the time of survey completion, 15 people (71.4%) were still attending the programme, 2 (9.5%) had completed the programme, and 3 (14.3%) left before the end of the programme. Those who left before programme completion reported work commitments (9.6% n=2) and family circumstances (4.8% n=1) as completion barriers. Of the 14 online group sessions (Extended Data-Appendix 3), the majority (81%, n=17) of respondents attended 6 or more sessions, 2 (9.5%) attended between 2–5 sessions, and 2 (9.5%) attended a single session.

### Demographics

The mean age of those who responded to the survey was 62 years (SD=11.6; range 36 to 82 years) and over half were men (57.1%, n=12). All self-identified their ethnicity as White. Fifty-two percent had advanced certificate or third level qualifications (n=11). More than half were retired (52.4%, n=11), one third were in paid employment (33.3%, n=7) with the remainder not working due to disability or looking after family or home (9.5%, n=2). No one described themselves as unemployed.

### Health factors

The majority of respondents reported that they rarely/never needed help understanding written material such as instructions or leaflets from the doctor or pharmacy (76.2%, n=16). Most described their general health as good (71.4%, n=15). The mean score on the MHI-5 (mental health) was 77.7 (SD=12.1). The mean on the EUROHIS-QOL 8-item (quality of life) was 31.38 (SD=3.8). All but 2 (90.5%, n=19) knew someone with diabetes while over half (52.4%, n=11) had a close family member with diabetes. Most had never previously participated in a programme to promote health (71.4%, n=15). Over half (57.2%, n=12) rated themselves as confident or very confident when it came to participating in an online DPP ( Table 1).

### Psychosocial factors

All respondents recognised they were at risk of diabetes and believed the programme could help them reduce their risk of diabetes. Most agreed it was important to manage this risk (95.2%, n=20) and 85.7% (n=18) agreed they could reduce the risk. The majority disagreed that diabetes was not serious (95.2% n=20), and all disagreed that the risk was too low to worry about. All but one respondent (95.2%, n=20) scored more than 50 in the Health Confidence Score and the mean self-efficacy score was 66.6%. The majority were happy with their current lifestyle (71.4%, n=15) and disagreed that going to the online programme required a lot of effort ( Table 2).

### Subgroup analysis

Men who took part in the survey were slightly older than the women (64 years vs 61 years). The men reported less confidence using online format with 33.3% (n=4) rating themselves as unconfident or very unconfident compared to 11.1% of the women (n=1) ( Table 2). A higher proportion of women (55.6%, n=5) than men (8.3%, n=1) had previously joined a group to improve health.

## Discussion

The aim of this study was to describe the demographic, psychosocial and health characteristics of participants in the pilot of the online NDPP in Ireland. The survey provided a unique opportunity to describe the profile of those who participated in an online DPP, and their perceptions of health and psychosocial factors that may affect participation. Findings provide a number of key insights in terms of participant characteristics. Firstly, men were well represented, secondly respondents had high levels of self-reported general health and finally they understood the seriousness of type 2 diabetes.

Those who responded to the survey were representative of the cohort (n=73) that participated in the pilot online NDPP ^20^ (Extended Data-Appendix 7). Over half the respondents in this study were men (57.1%). Men have historically been harder to reach with face-to-face DPPs ^21^. In a 2018 systematic review and meta-analysis of the impact of global diabetes prevention interventions, men represented only 28.8% of the 17,272 participants ^22^. Recent evidence indicates that a higher proportion of men attend digital DPPs (which include online programmes) than face-to-face programmes ^12, 23^ however, little is known about the reasons why. Further research should examine why this format is more attractive to men and what facilitated participation for those who rated their confidence with an online format as low.

NDPP participants who returned the survey considered themselves to be generally in good physical and mental health. Mental health scores were slightly above the national average for Ireland (77.7/100 vs 76/100) ^24^. Reported self-efficacy was higher than those who responded to an NHS DPP survey (67.08/100 Vs 64.6/100) ^7^. This is a positive finding as self-efficacy has been shown to favourably influence health behaviours such as the decision to attend digital DPPs ^10^. While it is possible that lifestyle changes made as part of the programme could have affected these scores it is also possible that the people with poorer health who were most at risk of diabetes did not attend the programme. Efforts need to be made to ensure people most at risk are recruited to DPPs to prevent a widening of health inequities ^25^. Successful strategies to improve attendance for people from diverse backgrounds such as using an extra session before the DPP involving motivational interviewing to increase risk awareness and problem solving around barriers should be considered ^26^.

Respondents in our study understood that type 2 diabetes was a serious disease, that they were at risk of developing it and that taking part in the DPP could help them to reduce that risk. These factors have been found to be important in uptake of the NHS DPP ^7^ and were found to affect motivation in a meta-synthesis of facilitators and barriers to lifestyle change in 2022 ^6^. It is possible that attending the programme influenced these beliefs. Further study is warranted to understand why the respondents in our study held these beliefs and if these beliefs could be encouraged in others at risk to improve participation.

## Strengths and limitations

This study has several strengths. We used an existing theoretically informed validated survey and modified it through discussion with the research team, health professionals, PPI and a university statistician. This ensured that the survey was appropriate for the Irish context. The study aligns with a positive deviance approach by investigating the characteristics of people who attended the online NDPP ^27^. This allows for greater understanding of the factors affecting the implementation of a successful healthcare practice, in this case attending the NDPP. Finally, and most importantly, it provides preliminary information on the first fully online NDPP, which can be used to improve participation in further roll out of the Irish NDPP and other online DPPs in the future and researchers will find this study helpful to understand participation when no other format such as face-to-face was available.

While the uptake of this survey was lower than the response to the NHS survey (31% vs 54%) the people who responded to the survey had a broad age range, a wide range of educational attainment, and there was good representation from both men and women. They may however have chosen to take part due to their positive views on the NDPP and additional insights may have been identified with a larger sample. The people attending the programme may have been the easiest to recruit, therefore further research should focus on understanding the views of those who declined to attend to improve participation in the future. Further research is also recommended with those who attended the initial assessment but did not attend further sessions to understand the factors affecting their decisions not to participate in the programme.

## Conclusion

This survey provides a snapshot into the demographic, health and psychosocial factors of participants in the online NDPP in Ireland. Participant’s perceptions may indicate potentially modifiable targets to increase participation in DPPs. Further qualitative research is planned to explore these factors in greater depth.

## Consent

Ethical approval for the study was given by the Clinical Research Ethics Committee of the Cork Teaching Hospitals (ref: ECM 4 (n) 10/8/21) Written informed consent to participate in the study and for findings to be published was obtained.

## Data Availability

The underlying data are not available for this study. The study participants did not give consent for their data to be shared in a public repository. The information leaflet they received stated that their data would be anonymised and reported in aggregate. This was deemed necessary as the study is reporting on a pilot programme with a small number of participants. The data contains information such as gender, age, ethnicity, educational level, number of classes attended and number of people in the household which could compromise confidentiality. Removing this data would compromise the usefulness of the dataset. Figshare: Characteristics of participants in the first fully online National Diabetes Prevention Programme: A quantitative survey http://doi.org/10.6084/m9.figshare.2412620426 ^28^. This project contains the following extended data: STROBE reporting guidelines (Appendix 1) Survey (Appendix 2) TIDieR description of the NDPP (Appendix 3) Invitation (Appendix 4) Participant Information Leaflet (Appendix 5) Consent form (Appendix 6) NDPP participant demographic information (Appendix 7) STROBE checklist for cross sectional studies was used (Appendix 1) Figshare: Characteristics of participants in the first fully online National Diabetes Prevention Programme: A quantitative survey http://doi.org/10.6084/m9.figshare.24126204 ^28^. Data are available under the terms of the Creative Commons Attribution 4.0 International license (CC-BY 4.0).
