# Supplementary material for: Characteristics of participants in the first fully online National Diabetes Prevention Programme: A quantitative survey
Source: HRB Open Res. 2025 Mar 6;6:61. Originally published 2023 Oct 18. [Version 4] doi: 10.12688/hrbopenres.13807.4 (PMC11808842; doi:10.12688/hrbopenres.13807.4)
Supplement: Peer review history

# Reviewer report: Reviewer response for version 3

**DOI:** 10.21956/hrbopenres.15429.r44229

**Published:** 2024-12-19

**Authors:** Lillian Madrigal

**Affiliations:** 1 Emory University, Atlanta, USA

Authors have addressed my comments.

Is the work clearly and accurately presented and does it cite the current literature?

Yes

If applicable, is the statistical analysis and its interpretation appropriate?

Yes

Are all the source data underlying the results available to ensure full reproducibility?

No source data required

Is the study design appropriate and is the work technically sound?

Yes

Are the conclusions drawn adequately supported by the results?

Yes

Are sufficient details of methods and analysis provided to allow replication by others?

Partly

Reviewer Expertise:

Implementation science, public health promotion programs, diabetes prevention and management, behavioral health, program evaluation

I confirm that I have read this submission and believe that I have an appropriate level of expertise to confirm that it is of an acceptable scientific standard.

# Reviewer report: Reviewer response for version 2

**DOI:** 10.21956/hrbopenres.15404.r43337

**Published:** 2024-12-17

**Authors:** Michael J Cannon

**Affiliations:** 1 Centers for Disease Control and Prevention, Atlanta, USA

This article describes demographic, psychosocial, and health characteristics of individuals who participated in a pilot online national diabetes prevention programme delivered via videoconferencing. This kind of information is important for understanding who is being reached by such programs, what factors might lead people to participate, and what sort of attitudes and motivation participants might have. The information was collected using validated survey instruments where applicable.

 Several findings indicate characteristics that might be targeted for participant recruitment, as well as health messages that may be needed to increase interest in the online program. They include that participants often had a family member with diabetes, were confident about joining an online program, believed that diabetes is important and that participating in the program could reduce their risk of developing diabetes.

 For understanding this study better, I would recommend adding a few sentences about recruitment in the section under Survey domains/Participation.

 How did recruitment work?

 How were people identified as eligible to participate?

 What percentage of people called agreed to participate and then actually participated?

 I realize this information may be in one of the cited reports, but a concise description here would really help the reader.

 The main limitation of the study is that the sample size was very small (N=22). As a result, it is hard to know how generalizable the results are. This, to me, is a serious limitation. Furthermore, the respondents only represented 30.5% of participants who were invited to participate (N=73), and they may differ in important ways from those who did not respond. As a result of these limitations, I do not have confidence that the findings can be applied more widely.

 For future research, in addition to having a larger sample of respondents, it would be great to have a better sense of how some of the measures differed for eligible individuals who declined participation in the NDPP (perhaps through surveys) and for all eligible participants in the country (perhaps using other data sources). This would give a stronger sense of what is different for those who decide to participate compared to those who do not.

Is the work clearly and accurately presented and does it cite the current literature?

Yes

If applicable, is the statistical analysis and its interpretation appropriate?

Yes

Are all the source data underlying the results available to ensure full reproducibility?

No

Is the study design appropriate and is the work technically sound?

Yes

Are the conclusions drawn adequately supported by the results?

Yes

Are sufficient details of methods and analysis provided to allow replication by others?

Yes

Reviewer Expertise:

I do research on the implementation of diabetes prevention and management programs.

I confirm that I have read this submission and believe that I have an appropriate level of expertise to state that I do not consider it to be of an acceptable scientific standard, for reasons outlined above.

# Response

**Published:** 2025-02-28

**Authors:** clair haseldine

**Affiliations:** UCC, Ireland

Dear Dr Cannon, 

 Thank you for you helpful review. I respond to all of the points you raised below. 

**1. For understanding this study better, I would recommend adding a few sentences about recruitment in the section under Survey domains/Participation**

 1. This information has been added in Version 3 in response to Reviewer 2 comments as detailed below. The page numbers and line numbers have been updated for the latest version (Version 4).

**How did recruitment work?**

**How were people identified as eligible to participate?**

 Further details have been added to “Survey Domains: Participation”

 Recruitment for the NDPP pilot was from existing dietetic waiting lists and by referrals from GPs who had been informed of the pilot.

 Page 5 lines 12, 13

**What percentage of people called agreed to participate and then actually participated?**

 Further clarification has been added to the section “Participants and procedure”

 Of the 158 people invited to attend the NDPP, 73 attended the initial assessment and 67 went on to attend at least one further session.

 Page 10 lines 5,6

**2. The main limitation of the study is that the sample size was very small (N=22). As a result, it is hard to know how generalizable the results are. This, to me, is a serious limitation. Furthermore, the respondents only represented 30.5% of participants who were invited to participate (N=73), and they may differ in important ways from those who did not respond. As a result of these limitations, I do not have confidence that the findings can be applied more widely.**

 2. We acknowledge the reviewer’s concerns about the sample size (N=22) and the response rate (30.5%). The number invited to take part in the pilot programme was small (N=73) which limited the overall number of survey respondents. Pilot programmes are often intentionally small to test the feasibility of implementing the programme before scale up and as a trial run of the intervention itself (Skivington et al, 2021). To maximise the response rate, we personalised the invite to the survey, included a €10 voucher to compensate the respondents for their time and included a stamped addressed envelope for the survey return, strategies shown to be effective in a Cochrane systematic review (Edwards, et al, 2007).

 References

 Skivington K, Matthews L, Simpson SA, Craig P, Baird J, Blazeby JM, Boyd KA, Craig N, French DP, McIntosh E, Petticrew M. A new framework for developing and evaluating complex interventions: update of Medical Research Council guidance. bmj. 2021 Sep 30;374.

 Edwards PJ, Roberts IG, Clarke MJ, DiGuiseppi C, Wentz R, Kwan I, Cooper R, Felix L, Pratap S. Methods to increase response rates to postal questionnaires. Cochrane Database of systematic reviews 2007, issue 2. Art No : MR000008

 The generalisability of small sample size and response rate are discussed further in the strengths and limitations section:

 The uptake of this survey was lower than the response to the NHS survey (31% vs 54%) however, the people who responded to the survey had a broad age range, a wide range of educational attainment, and there was good representation from both men and women. While the small sample size and response rate may limit the generalisability of this research note, the findings provide an update to researchers and DPP providers about the online pilot of an NDPP and the emerging evidence on the characteristics of those invited to attend.

 Page 13 lines 25 - 30

**3. For future research, in addition to having a larger sample of respondents, it would be great to have a better sense of how some of the measures differed for eligible individuals who declined participation in the NDPP (perhaps through surveys) and for all eligible participants in the country (perhaps using other data sources). This would give a stronger sense of what is different for those who decide to participate compared to those who do not.**

 3. Originally, we had hoped to survey the people who declined to attend but this access to this data was not possible. The recommendations for further research have been added to the strengths and limitations section as suggested:

 The people attending the programme may have been the easiest to recruit, or they may have differed in important ways from those who chose to respond, therefore further research (such as survey or other data sources) should focus on understanding the views of those who declined to attend to improve participation in the future.

 Page 13 lines 33 and 34

# Reviewer report: Reviewer response for version 2

**DOI:** 10.21956/hrbopenres.15404.r43334

**Published:** 2024-12-02

**Authors:** Lillian Madrigal

**Affiliations:** 1 Emory University, Atlanta, USA

This paper presents the results of a survey of participants in Ireland who had attended an initial assessment to participate in a pilot online synchronous version of the Irish National Diabetes Prevention Program. The study aims are to describe the demographic, psychosocial and health characteristics of participants in this online pilot.

 The evaluation methods appear to be sound for the aims of the study and results are clearly written. The findings of this work are useful for others implementing DPPs as well as other health promotion programs as they can replicate the surveys to assess demographic, psychosocial and health characteristics of participants in their populations. As someone who implements DPPs myself, I find the psychosocial factors in particular useful for understanding awareness and motivation to participate in the program. As the authors also point out it is nice to see an evaluation with a good representation of men in the cohort as men typically have lower participation rates in these programs.

 I have one revision request for the methods. I am unclear who the initial pool of eligible participants was. Under "Survey Domains: Participation" it says "educators invited people eligible" - who are these educators? where are the people from? Was a list of eligible candidates pulled from the HSE? What was the denominator to get to the 73 participants for the initial assessment? I believe more details are needed here to understand any bias in the recruitment and participants selected.

 Furthermore, a big challenge with DPPs is the "conversion rate" meaning the percent of individuals who are identified as eligible for these programs who actually enroll. I think that would be helpful to understand as many in the field as trying to assess if recruitment into programs is more successful for online vs distance vs in-person programs. Some comment on this might also be useful in the discussion.

Is the work clearly and accurately presented and does it cite the current literature?

Yes

If applicable, is the statistical analysis and its interpretation appropriate?

Yes

Are all the source data underlying the results available to ensure full reproducibility?

No source data required

Is the study design appropriate and is the work technically sound?

Yes

Are the conclusions drawn adequately supported by the results?

Yes

Are sufficient details of methods and analysis provided to allow replication by others?

Partly

Reviewer Expertise:

Implementation science, public health promotion programs, diabetes prevention and management, behavioral health, program evaluation

I confirm that I have read this submission and believe that I have an appropriate level of expertise to confirm that it is of an acceptable scientific standard, however I have significant reservations, as outlined above.

# Response

**Published:** 2024-12-13

**Authors:** clair haseldine

**Affiliations:** UCC, Ireland

Dear Dr Madrigal,

 Thank you for your helpful comments. Please see the responses below to each point raised.

**I have one revision request for the methods. I am unclear who the initial pool of eligible participants was. Under "Survey Domains: Participation" it says "educators invited people eligible"**

 Further details have been added to “Survey Domains: Participation”. Full details of the NDPP are available in Extended data – Appendix 3

**Who are these educators?**

 Educators (dieticians in the HSE) invited people eligible to participate in the NDPP by phone.

 Page 5 line 9

**Where are the people from? Was a list of eligible candidates pulled from the HSE?**

 Recruitment for the NDPP pilot was from existing dietetic waiting lists and by referrals from GPs who had been informed of the pilot.

 Page 5 lines 8, 9

**What was the denominator to get to the 73 participants for the initial assessment?** 

 Further clarification has been added to the section “Participants and procedure”

 Of the 158 people invited to attend the NDPP, 73 attended the initial assessment and 67 went on to attend at least one further session.

 Page 10 lines 5,6

**Furthermore, a big challenge with DPPs is the "conversion rate" meaning the percent of individuals who are identified as eligible for these programs who actually enroll. I think that would be helpful to understand as many in the field as trying to assess if recruitment into programs is more successful for online vs distance vs in-person programs. Some comment on this might also be useful in the discussion.**

 These are important points however the pilot was testing the feasibility of implementing the programme and it was not designed to provide definite data on recruitment. Further research is needed to understand if recruitment is more successful for online Vs distance Vs in-person programmes.

 To acknowledge that this study may be useful to researchers investigating the conversion rates with different modes of delivery I have added the following comments to the strengths and limitations:

 “…and researchers will find this study helpful to understand participation when no other format such as face-to-face was available.”

 Page 13, lines 10 and 11

# Reviewer report: Reviewer response for version 2

**DOI:** 10.21956/hrbopenres.15404.r43309

**Published:** 2024-11-25

**Authors:** Rhiannon Hawkes

**Affiliations:** 1 University of Manchester, Manchester, UK

Thank you for addressing my comments, I have no further comments to make. I'm looking forward to hearing about future work from the research team on the Irish NDPP.

Is the work clearly and accurately presented and does it cite the current literature?

Yes

If applicable, is the statistical analysis and its interpretation appropriate?

Yes

Are all the source data underlying the results available to ensure full reproducibility?

Yes

Is the study design appropriate and is the work technically sound?

Yes

Are the conclusions drawn adequately supported by the results?

Yes

Are sufficient details of methods and analysis provided to allow replication by others?

Yes

Reviewer Expertise:

Diabetes prevention programmes, behaviour change, health psychology

I confirm that I have read this submission and believe that I have an appropriate level of expertise to confirm that it is of an acceptable scientific standard.

# Reviewer report: Reviewer response for version 1

**DOI:** 10.21956/hrbopenres.15110.r40518

**Published:** 2024-06-20

**Authors:** Rhiannon Hawkes

**Affiliations:** 1 University of Manchester, Manchester, UK

This is a well-written paper describing findings from a survey distributed to participants of pilot online diabetes prevention programme in Ireland. This is particularly novel as Ireland is the first to pilot a national DPP that is online only and delivered to a group synchronously. Findings from this programme of work will therefore be of interest to other countries developing their own versions of diabetes prevention initiatives. Thank you for the opportunity to review this paper, and I look forwards to seeing further work from the research team to see how the Irish NDPP progresses. The following comments are some minor suggestions for consideration:

 1. There are some instances throughout the abstract and manuscript where there needs to be a space between a word and opening bracket, for example: “the Health Service Executive(HSE)”, “HSE(national publicly funded healthcare system)”.

 2. The abbreviations NDPP and HSE are defined in the abstract, but these should be defined again at first mention in the main manuscript.

 3. At the start of the introduction the authors could consider adding in a brief sentence explaining why type 2 diabetes is a growing problem (e.g., complications and costs to the health service) to further set the scene for readers.

 4. Prior to the NDPP being piloted, was Ireland offering any other diabetes prevention initiatives? An extra sentence in the introduction with some context might be useful for readers.

 5. It is mentioned that participants attend an initial assessment that was followed by 14 sessions of the NDPP. Appendix 3 is particularly useful here, but perhaps it could be briefly explained in the paper what the initial assessment might entail for readers who are less familiar with DPPs? For example, was the initial assessment solely to determine the eligibility of participants for the programme, or were baseline measures also taken? How long did the initial assessment last? Was it online or in-person? Does this include onboarding or is that a separate session?

 6. In Appendix 3 of the TIDieR checklist, could the abbreviation CNS be clarified?

 7. It is mentioned that the survey was piloted with a patient and public involvement group, which is commendable and an example of best practice. Could there be some more information about how PPI shaped this research? E.g., what were the characteristics of the group? (Males/females, ages, were these people at risk of or living with type 2 diabetes?) What feedback did they provide on the survey and what feedback was incorporated into the final version of the survey?

 8. The survey response rate was 31%, and although this was lower than similar studies conducted (e.g., response to the NHS DPP survey), it is noted that respondents were representative of the 73 people in the cohort who participated in the NDPP. Out of curiosity I was wondering whether the research team had considered also offering the survey as an online format (e.g., via Qualtrics or another platform), which might have increased the response rate, given that respondents were taking part in an online programme? Were any reminder letters sent out? Perhaps these could be future considerations if a similar evaluation is conducted on the NDPP (if the programme is continuing?)

 9. Limitations – is there the potential that the 73 people who participated in the pilot NDPP were the ‘low hanging fruit’, i.e. the people who were most likely to participate in the programme in the first place? So this study is useful for understanding how to improve participation in the ‘engaged’ participants, but less useful for understanding why people don’t participate. There is mention of understanding the views of those who declined to participate in the NDPP, but there could also be further mention of understanding reasons for non-participation of those who attended the initial assessment but did not attend any online sessions of the NDPP.

 10. What are the plans for the NDPP in Ireland now that the pilot programme has finished? If the programme is continuing, what will the programme look like and is there any further evaluation work planned?

Is the work clearly and accurately presented and does it cite the current literature?

Yes

If applicable, is the statistical analysis and its interpretation appropriate?

Yes

Are all the source data underlying the results available to ensure full reproducibility?

Yes

Is the study design appropriate and is the work technically sound?

Yes

Are the conclusions drawn adequately supported by the results?

Yes

Are sufficient details of methods and analysis provided to allow replication by others?

Yes

Reviewer Expertise:

Diabetes prevention programmes, behaviour change, health psychology

I confirm that I have read this submission and believe that I have an appropriate level of expertise to confirm that it is of an acceptable scientific standard, however I have significant reservations, as outlined above.

# Response

**Published:** 2024-11-19

**Authors:** clair haseldine

**Affiliations:** UCC, Ireland

Dear Reviewer,

 Thank you for your helpful review. We made every effort to address all the points raised. The changes are highlighted in red in the revised text.

**1.There are some instances throughout the abstract and manuscript where there needs to be a space between a word and opening bracket, for example: “the Health Service Executive(HSE)”, “HSE(national publicly funded healthcare system)”.**

 Thank you for highlighting this. I have corrected the issue with spacing throughout the manuscript.

**2. The abbreviations NDPP and HSE are defined in the abstract, but these should be defined again at first mention in the main manuscript.**

 I have now defined the abbreviations at first mention in the main manuscript:

 Page 4, line 19

 Page 4, line 20

**3. At the start of the introduction the authors could consider adding in a brief sentence explaining why type 2 diabetes is a growing problem (e.g., complications and costs to the health service) to further set the scene for readers.**

 I have now included the following line on the growing problem of type 2 diabetes  to set the scene at the start of the introduction.

 Page 3, lines 2, 3

 “Type 2 diabetes is a growing global health problem which can result in serious chronic health complications for individuals and places a financial burden on health systems.”

 Reference: International Diabetes Federation. IDF Diabetes Atlas, 10th edn. Brussels, Belgium: 2021. Available at https://www.diabetesatlas.org

**4. Prior to the NDPP being piloted, was Ireland offering any other diabetes prevention initiatives? An extra sentence in the introduction with some context might be useful for readers.**

 I have added the following information on diabetes prevention initiatives that were available before the introduction of the NDPP.

 Page 4, lines 21, 22

 “Prior to this there was very limited availability to short prediabetes courses that were not standardised.”

**5. It is mentioned that participants attend an initial assessment that was followed by 14 sessions of the NDPP. Appendix 3 is particularly useful here, but perhaps it could be briefly explained in the paper what the initial assessment might entail for readers who are less familiar with DPPs? For example, was the initial assessment solely to determine the eligibility of participants for the programme, or were baseline measures also taken? How long did the initial assessment last? Was it online or in-person? Does this include onboarding or is that a separate session?**

 I have added details about the initial assessment.

 Page 5 lines 13 - 15

 “NDPP participants attended an initial assessment (in person, online or on the phone to have baseline measurements recorded, to receive a personal plan for lifestyle change and onboarding for the online session), and this was followed by 14 sessions of the NDPP (Extended Data-Appendix 3).”

 Note: Under extended data in the paper the appendices were labelled incorrectly – this has now been corrected.

 Page 14, lines 20 - 22

**6. In Appendix 3 of the TIDieR checklist, could the abbreviation CNS be clarified?**

 The abbreviation CNS has been clarified to Clinical Nurse Specialist  in the TIDieR checklist in Appendix 3.

**7. It is mentioned that the survey was piloted with a patient and public involvement group, which is commendable and an example of best practice. Could there be some more information about how PPI shaped this research? E.g., what were the characteristics of the group? (Males/females, ages, were these people at risk of or living with type 2 diabetes?) What feedback did they provide on the survey and what feedback was incorporated into the final version of the survey?**

 Thank you for these suggestions. I have included further information on the contribution of the PPI panel however I have not included their demographic or medical history as I do not have their permission to do so.  

 Page 5, lines 3 - 6:

 “The PPI group consisted of 10 people with an interest in diabetes prevention. They advised on recruitment and the wording of questions. Based on their feedback on recruitment, the cover letters used the participant’s name rather than a generic address.”

**8. The survey response rate was 31%, and although this was lower than similar studies conducted (e.g., response to the NHS DPP survey), it is noted that respondents were representative of the 73 people in the cohort who participated in the NDPP. Out of curiosity I was wondering whether the research team had considered also offering the survey as an online format (e.g., via Qualtrics or another platform), which might have increased the response rate, given that respondents were taking part in an online programme? Were any reminder letters sent out? Perhaps these could be future considerations if a similar evaluation is conducted on the NDPP (if the programme is continuing?)**

 Thank you for those suggestions.

 We had initially hoped to also survey people who declined to attend, therefore we developed the postal survey so as not to exclude those who may not have been comfortable with the online format.

 As the research team did not have access to the participant’s details the educators sent out the surveys on our behalf. Due to time constraints a reminder letter was not sent.

 I agree that your suggestions could be very helpful in the future if another evaluation is conducted.

**9. Limitations – is there the potential that the 73 people who participated in the pilot NDPP were the ‘low hanging fruit’, i.e. the people who were most likely to participate in the programme in the first place? So this study is useful for understanding how to improve participation in the ‘engaged’ participants, but less useful for understanding why people don’t participate. There is mention of understanding the views of those who declined to participate in the NDPP, but there could also be further mention of understanding reasons for non-participation of those who attended the initial assessment but did not attend any online sessions of the NDPP.**

 I have acknowledged these limitations and the need for further research with those who declined to participate.

 Page 13, lines 31 - 36

 “The people attending the programme may have been the easiest to recruit, therefore further research should focus on understanding the views of those who declined to attend to improve participation in the future. Further research is also recommended with those who attended the initial assessment but did not attend further sessions to understand the factors affecting their decisions not to participate in the programme.”

**10. What are the plans for the NDPP in Ireland now that the pilot programme has finished? If the programme is continuing, what will the programme look like and is there any further evaluation work planned?**

 At the time of this survey the pilot was ongoing. On completion of the pilot in 2022 the NDPP began a phased nationwide roll out and since mid-2024 has offered a face-to-face programme in addition to the online format. I am not aware of any further evaluation of the programme at this time.
